# Urinary Peptidomic Signature for Noninvasive Diagnosis of Alport Syndrome in Children

**DOI:** 10.1016/j.ekir.2026.107093

**Published:** 2026-09-25

**Authors:** Julia Byrska, Jan Boeckhaus, Kleanthi Voutsadaki, Benjamin Breuil, Eléonore Delecroix, Sheila Murunga, Bénédicte Buffin-Meyer, Brian Becknell, Daryl McLeod, Kathrin Burgmaier, Max C. Liebau, Franz Schaefer, Justyna Siwy, Harald Mischak, Margarita Kondyli, Stéphane Decramer, Maria D. Sanchez-Niño, Alberto Ortiz, Oliver Gross, Joost P. Schanstra

**Affiliations:** 1 Department of Nephrology and Hypertension, Health Research Institute-Fundación Jiménez Díaz University Hospital, Universidad Autónoma de Madrid, Madrid, Spain; 2 Clinic of Nephrology and Rheumatology, University Medical Center Goettingen, Goettingen, Germany; 3 Institut National de La Santé et De La Recherche Médicale (INSERM), UMR 1297, Institute of Metabolic and Cardiovascular Disease, Toulouse, France; 4 University of Toulouse, Toulouse, France; 5 Division of Pediatric Nephrology, Department of Pediatrics, Faculty of Medicine, Heidelberg University, Heidelberg, Germany; 6 Kidney and Urinary Tract Center, The Abigail Wexner Research Institute at Nationwide Children’s Hospital, Columbus, Ohio, USA; 7 Division of Nephrology and Hypertension, Nationwide Children’s Hospital, Columbus, Ohio, USA; 8 Department of Pediatrics, Faculty of Medicine, University Hospital Cologne and University of Cologne, Cologne, Germany; 9 Faculty of Applied Healthcare Science, Deggendorf Institute of Technology, Deggendorf, Germany; 10 Department of Pediatrics, University Hospital Aachen, RWTH Aachen University, Aachen, Germany; 11 Mosaiques Diagnostics, Hannover, Germany; 12 Institute for Molecular Cardiovascular Research (IMCAR), RWTH Aachen University Hospital, Aachen, Germany; 13 Department of Pediatric Internal Medicine, Rheumatology and Nephrology, Toulouse University Hospital, Toulouse, France; 14 Centre de Référence Des Maladies Rénales Rares du Sud-Ouest (SORARE), Toulouse University Hospital, Toulouse, France; 15 RICORS2040-renal, Madrid, Spain; 16 Departamento de Farmacología, Facultad de Medicina, Universidad Autónoma de Madrid, Madrid, Spain; 17 Departamento de Medicina, Facultad de Medicina, Universidad Autónoma de Madrid, Madrid, Spain

**Keywords:** Alport syndrome, pediatric nephrology, urinary peptidomics

## Abstract

**Introduction:**

Early diagnosis of Alport syndrome (AS) in children remains challenging because early manifestations are nonspecific. Some clinically suspected cases remain genetically unresolved because no disease-causing variant has been identified or the variants identified are of uncertain significance. Urinary peptidomics is a promising noninvasive biomarker approach. We aimed to develop and validate a urinary peptidomic signature for pediatric AS.

**Methods:**

Urinary peptide profiles generated by capillary electrophoresis-mass spectrometry were analyzed in 282 urine samples from 57 children with AS (80.7% had X-linked disease, mean age was 10 ± 4 years, 4% were female, and mean estimated glomerular filtration rate [eGFR] was 128 ± 31 ml/min per 1.73 m^2^) enrolled in EARLY PRO-TECT trial, along with 651 samples from pediatric kidney disease cohorts and healthy controls. The data set was randomly divided into discovery (70%) and validation (30%) cohorts. Differential peptides identified by Wilcoxon testing with Benjamini-Hochberg adjustment were used to develop a support vector machine classifier. Performance was assessed by cross-validation and application to the validation cohort.

**Results:**

A total of 151 discriminatory peptides were identified. Sequence information was available for 87 peptides used for classifier generation; 60.9% were collagen-derived. In validation, the classifier achieved an area under the receiver operating characteristic curve (AUC) of 0.999 (95% confidence interval [CI], 0.997–1.000), with 100% sensitivity, 97.4% specificity, and 98.2% accuracy. After adjustment for age, sex, and eGFR, performance remained high (adjusted AUC, 0.918 [95% CI, 0.884–0.951]). Longitudinal analyses demonstrated stable classifier scores over up to 3 years.

**Conclusion:**

We developed and validated an accurate urinary peptidomic classifier for noninvasive diagnosis of pediatric AS. The signature may complement genetic testing and support earlier recognition of AS.

## Graphical abstract

**Figure:**
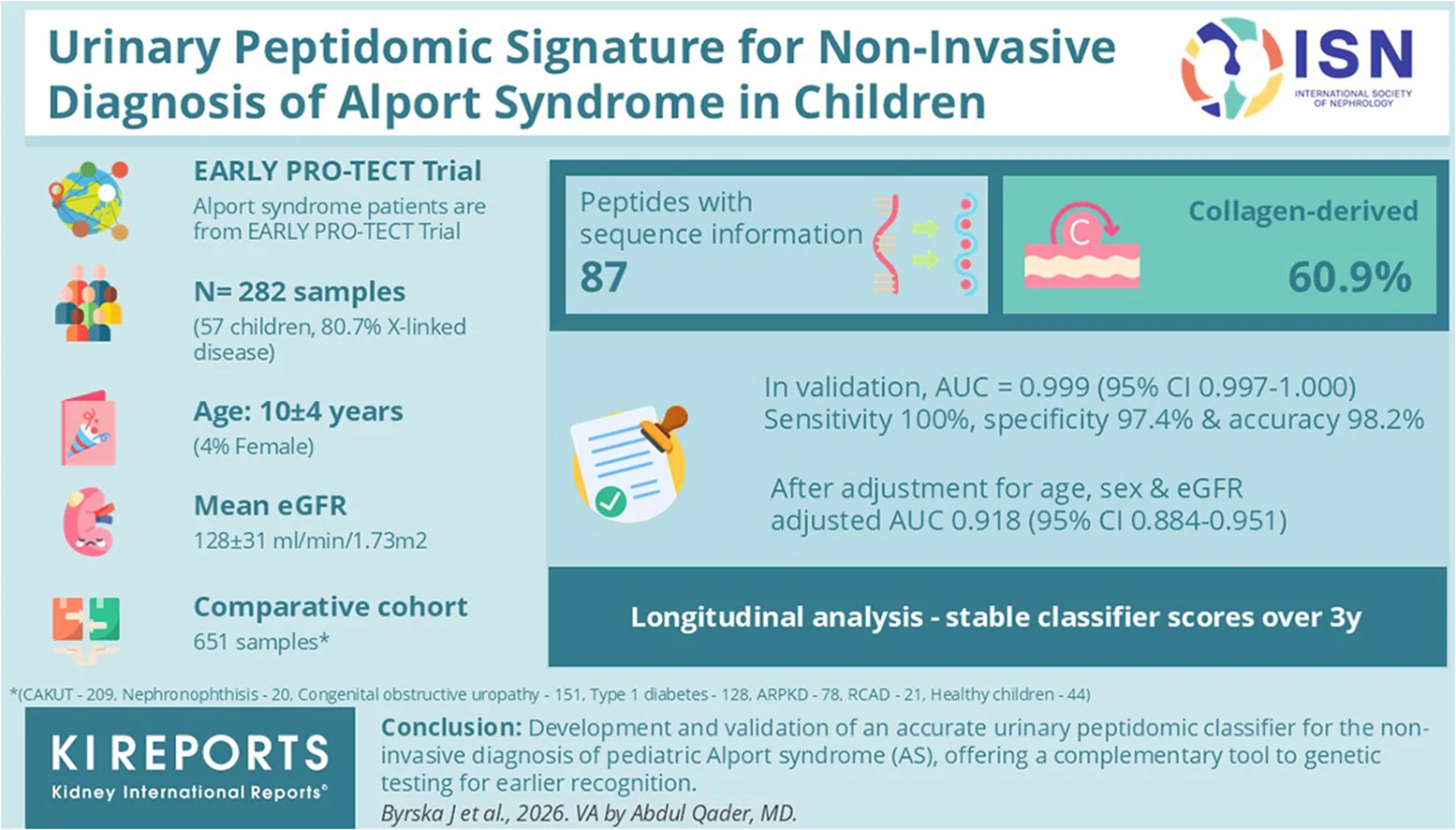

Inherited kidney disease and congenital abnormalities of the kidney and urinary tract (CAKUT), which may have a genetic cause, are the second most common causes of prevalent kidney replacement therapy in Europe, after glomerular diseases, and the most common cause in children.^1^ However, hereditary nephropathy (subtype unknown) is the second most common inherited cause after autosomal dominant polycystic kidney disease, suggesting widespread underdiagnosis of specific inherited nephropathies. AS is an inherited kidney disease caused by pathogenic variants in the *COL4A3*, *COL4A4*, or *COL4A5* genes, leading to abnormalities in the structure of type IV collagen and progressive glomerular basement membrane damage. AS has a variable phenotype that may range from isolated hematuria to progressive chronic kidney disease (CKD), hearing loss, and eye abnormalities, depending on the inheritance pattern and genetic variants.^2^

Early diagnosis of AS is important in childhood because it can guide follow-up strategies, enable family screening, and support early initiation of nephroprotective treatment, including renin-angiotensin system inhibitors, which can delay disease progression.^3^ However, diagnosis in children remains challenging. Early manifestations such as persistent microscopic hematuria are nonspecific and frequently overlap with other pediatric kidney disorders, which complicates diagnosis.^4^ Although genetic testing is now central to the diagnostic approach, current sequencing strategies do not detect all causative variants, leaving a subset of clinically suspected cases without a confirmed molecular diagnosis. A recent review highlighted that up to 20% of individuals with suspected AS did not have a disease-causing variant identified through genetic testing.^5^ This unresolved proportion is not attributable to a single testing strategy and includes both cases in which no candidate variant is detected and cases in which an identified variant remains of uncertain significance. Potential explanations include technical limitations of whole-exome sequencing, difficulties in detecting or interpreting noncanonical splice variants, including deep-intronic and synonymous changes, mosaicism, and phenocopies caused by other kidney diseases.^5^ Kidney biopsy may also contribute to the diagnosis of AS, particularly when genetic findings are negative, inconclusive, or discordant with the clinical phenotype. Electron microscopy and collagen IV immunostaining may provide additional diagnostic information, although biopsy is invasive, and histologic findings, particularly on light microscopy, may be nonspecific.^3^ These limitations have increased interest in noninvasive urine-based approaches that could support earlier and more accessible detection of AS.

Recent pediatric data support the value of urine-based assessment. Urine screening at the age of 3 years was shown to identify a substantial proportion of AS cases early, with many children already eligible for renin-angiotensin system inhibitor treatment at the time of detection.^6^ Beyond conventional urinalysis, specific urinary proteins, peptides, and proteomic biomarkers are increasingly recognized as valuable tools for the early detection of kidney injury and for improving diagnostic precision beyond traditional clinical markers. Recent studies demonstrated that urinary protein biomarkers can identify early kidney damage in children with AS independently of albuminuria and inflammation.^7^ In parallel, urinary peptidomics has emerged as a promising approach in chronic kidney disease, with growing evidence that disease-specific molecular signatures may improve disease classification and diagnostic accuracy.^8^

In pediatric nephrology, disease-specific urinary peptidomic signatures have already been successfully developed for several kidney disorders. In renal cysts and diabetes syndrome (RCAD), a panel of 146 discriminatory peptides enabled accurate differentiation from relevant comparator controls, while also providing biological insight into disease-associated extracellular matrix remodeling.^9^ Similarly, in autosomal recessive polycystic kidney disease (ARPKD), a 77-peptide signature demonstrated excellent diagnostic performance in validation cohorts, with a predominance of collagen-derived peptides reflecting underlying structural kidney injury.^10^ However, despite these advances, no disease-specific urinary peptidomic signature has yet been established for the diagnosis of AS in children.

In this study, we aimed to develop and validate a disease-specific urinary peptidomic signature for the noninvasive diagnosis of AS in children and to explore its potential as a complementary diagnostic tool alongside genetic testing and, when clinically indicated, kidney biopsy, particularly in cases with inconclusive or negative molecular findings.

## Methods

### Study Population

A total of 282 urine samples from 57 pediatric patients with AS, derived from the EARLY PRO-TECT trial^11^ (NCT01485978) were included in the analysis. In the EARLY PRO-TECT trial, the diagnosis of AS was established based on kidney biopsy in the patient or an affected relative, or molecular genetic testing, together with clinical diagnostic criteria including hematuria, family history of kidney disease, ocular abnormalities, and sensorineural hearing loss. Genetic variants were subsequently reassessed according to American College of Medical Genetics and Genomics criteria, with patients carrying pathogenic or likely pathogenic *COL4A3–COL4A5* gene variants with a compatible genotype classified as genetically solved. According to the genetic classification reported in the EARLY PRO-TECT cohort publication,^12^ among the 57 pediatric patients included in the present analysis, 49 (86%) were classified as genetically solved and 8 (14%) as genetically unresolved, despite having a clinical diagnosis of AS.

The comparative cohorts comprised 651 samples, including 209 samples from 209 pediatric patients with CAKUT from the Cardiovascular Comorbidity in Children with Chronic Kidney Disease (4C) Study^13^ (NCT01046432), 20 samples from 20 pediatric patients with nephronophthisis from the same study, 151 samples from 151 pediatric patients with congenital obstructive uropathy from the CKD in Children Study,^14^ 128 samples from 128 pediatric patients with type 1 diabetes from the PeeDIAB study (DC 2008-452), 78 samples from 40 pediatric patients from the international ARPKD registry study (ARegPKD),^15^ 44 samples from 44 healthy children from the Human Urinary Proteome Database,^16^ and 21 samples from 21 pediatric patients with RCAD from the same database.

Sex was defined according to the male/female classification recorded in the original clinical datasets. eGFR was calculated using different equations depending on the study cohort. For participants from the Cardiovascular Comorbidity in Children with Chronic Kidney Disease (4C) Study, the EARLY PRO-TECT trial, PeeDIAB study, and samples from the Human Urinary Proteome Database, eGFR was estimated using the Schwartz formula.^17^ For individuals in the CKD in children Study, eGFR was calculated based on the U25 equation,^18^ whereas for individuals with ARPKD, eGFR was derived using the full age spectrum equation.^19^ Age and eGFR were calculated at the sample or visit level due to longitudinal sampling. An overview of the study design and classifier development workflow is presented in Figure 1.

**Figure 1:**
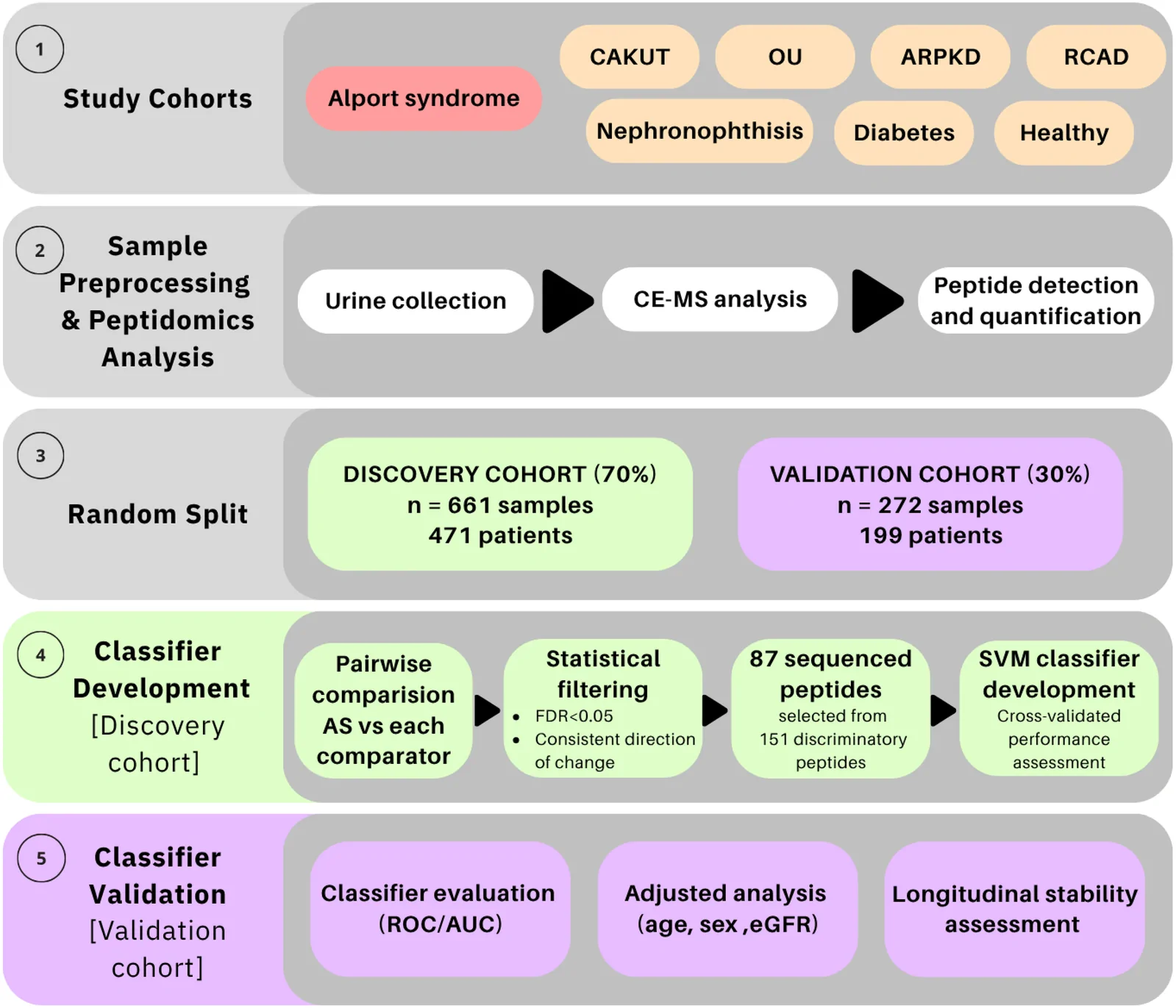
Study design and workflow for development and validation of the pediatric urinary peptidomic classifier for Alport syndrome. ARPKD, Autosomal Recessive Polycystic Kidney Disease; AUC, area under the curve; CAKUT, Congenital Anomalies of the Kidney and Urinary Tract; CE-MS, capillary electrophoresis-mass spectrometry; eGFR, estimated glomerular filtration rate; FDR, false discovery rate; OU, obstructive uropathies; RCAD, Renal cysts and diabetes syndrome; ROC, receiver operating characteristic.

### Data Preprocessing

Urinary peptide profiles were generated from individual urine samples using capillary electrophoresis coupled to mass spectrometry. Sample preparation, capillary electrophoresis coupled to mass spectrometry measurements, and data processing were conducted according to standardized ISO13485-compliant procedures. Peptide sequencing was performed using tandem mass spectrometry (MS/MS). Detailed methodological information is provided in the Supplementary Methods.

### Statistical Analysis

Statistical analysis was performed in R (version 4.4.3).^20^ Analysis was limited to peptides detected in at least 30% of samples in each cohort. For visualization of global urinary peptide profiles, t-distributed stochastic neighbor embedding (t-SNE) was performed using the Rtsne package (version 0.17)^21^ after principal component analysis, using the first 50 principal components and a perplexity of 30. To develop and evaluate the classifier and to avoid data leakage because of longitudinal sampling, the dataset was randomly divided at the patient level into a discovery cohort (70%) and an independent validation cohort (30%), ensuring that all samples from a given patient were assigned to only 1 dataset.

All peptide selection procedures were performed in the discovery cohort. Pairwise comparisons were conducted between the AS group and each of the other groups (7 comparisons in total), and for each peptide, log2 fold change and false discovery rate were calculated using the Wilcoxon rank-sum test, with *P*-values adjusted for multiple testing according to the Benjamini–Hochberg false discovery rate procedure.^22^ Peptides were filtered to retain only those showing a consistent direction of change across all comparisons (i.e., consistently increased or decreased in AS vs. all other groups) and meeting the significance threshold of false discovery rate < 0.05 in each comparison. Among the resulting discriminatory peptides, only peptides with available sequence information were retained and used for classifier construction.

### Classifier Generation

Based on the selected sequenced peptides, a support vector machine model was constructed to perform binary classification. The model was trained in the discovery cohort to discriminate patients with AS from the combined control group. To limit the impact of overfitting, classifier performance in the discovery cohort was estimated using cross-validation.

The support vector machine model was developed using MosaCluster software (version 1.6.5) with a radial basis function kernel. Optimal hyperparameters for the model were identified through parameter tuning. The resulting classifier assigned each sample a continuous classification score reflecting its position relative to the decision boundary in multidimensional feature space. Classifier performance was evaluated using the AUC with 95% CI using the pROC package (version 1.19.0.1).^23^ The optimal classification threshold was determined from the cross-validated scores in the discovery cohort using the Youden index and applied unchanged to the independent validation cohort.

### Adjustment for Demographic and Clinical Variables

To assess whether classifier performance was influenced by demographic or clinical differences between the cohorts, classifier scores in the validation set were adjusted for age, sex, and eGFR using linear regression, with classifier score included as the dependent variable and age, sex, and eGFR included as covariates. Residualized adjusted scores derived from this model were evaluated by receiver operating characteristic curve analysis, and the AUC was calculated. This adjusted analysis was performed as a sensitivity analysis and was not used to redefine the diagnostic threshold. A second model incorporating demographic and clinical variables directly was not developed because the primary aim was to evaluate the diagnostic performance of the urinary peptidomic classifier as a standalone molecular test, with covariate adjustment used only to assess potential confounding.

### Longitudinal Analyses

Longitudinal analyses were performed in pediatric patients with AS from the validation cohort using repeated urine samples collected during follow-up to assess classifier stability over time and potential associations with angiotensin-converting enzyme inhibitor (ACEi) treatment and disease progression. ACEi treatment status was recorded at each visit, whereas progression status was defined at the patient level according to the EARLY PRO-TECT study criteria. Linear mixed-effects models with patient-specific random intercepts were used to account for repeated measurements within individuals.

## Results

### Characteristics of the Study Population

All pediatric patients with AS showed preserved kidney function, with a mean eGFR of 128 ± 31 ml/min per 1.73 m^2^, comparable with the diabetes (134 ± 23 ml/min per 1.73 m^2^) and RCAD (115 ± 19 ml/min per 1.73 m^2^) cohorts. In contrast, markedly lower eGFR values were observed in the CAKUT (30 ± 12 ml/min per 1.73 m^2^), obstructive uropathy (49 ± 18 ml/min per 1.73 m^2^), ARPKD (69 ± 35 ml/min per 1.73 m^2^), and nephronophthisis (28 ± 9 ml/min per 1.73 m^2^) cohorts. The cohorts also differed in age distribution and sex composition. Mean age ranged from 6 ± 4 years in the RCAD cohort to 12 ± 3 years in nephronophthisis, and the proportion of female participants ranged from 4% in AS to 53% in ARPKD (Table 1 and Supplementary Table S1). The strong male predominance within the AS cohort was consistent with the high proportion of X-linked disease. Among the 57 pediatric patients with AS, 46 had X-linked disease, 9 had autosomal recessive AS, and 2 had an undefined mode of inheritance.

**Table 1:** Clinical and demographic characteristics of the discovery and validation cohorts used for classifier development and evaluation

Characteristics	Discovery cohort				Validation cohort			
	Alport syndrome	Non–AS kidney disease^a^	Type 1 diabetes	Healthy	Alport syndrome	Non–AS kidney disease^b^	Type 1 Diabetes	Healthy
Samples, *n*	201	339	90	31	81	140	38	13
Patients, *n*	40	310	90	31	17	131	38	13
Age, yr, mean (SD)	10 (4)	10 (4)	10 (4)	10 (5)	11 (4)	10 (5)	10 (4)	11 (5)
Female, %	5	26	53	32	0	26	50	46
eGFR (ml/min per 1.73 m^2^), mean (SD)	131 (30)	47 (29)	132 (22)	NA	121 (34)	44 (26)	138 (25)	NA

ARPKD, Autosomal Recessive Polycystic Kidney Disease; CAKUT, Congenital Anomalies of the Kidney and Urinary Tract; eGFR, estimated glomerular filtration rate; NA, not applicable; OU, obstructive uropathies; RCAD, Renal cysts and diabetes syndrome; SD, standard deviation.

a CAKUT (147 samples/147 patients), OU (106 samples/106 patients), ARPKD (57 samples/28 patients), RCAD (15 samples/15 patients), and Nephronophthisis (14 samples/14 patients).

b CAKUT (62 samples/62 patients), OU (45 samples/45 patients), ARPKD (21 samples/12 patients), RCAD (6 samples/6 patients), and Nephronophthisis (6 samples/6 patients).

### Definition of Classifier

To ensure robust representation across the cohort, only peptides among the 20,192 observed peptides that were detected in at least 30% of samples were retained for analysis, resulting in a final dataset of 7451 peptides. Unsupervised dimensionality reduction of urinary peptide profiles showed distinct clustering of the study cohorts, with AS samples separating from the other groups, suggesting that AS is associated with a distinct urinary peptidomic profile (Figure 2).

**Figure 2:**
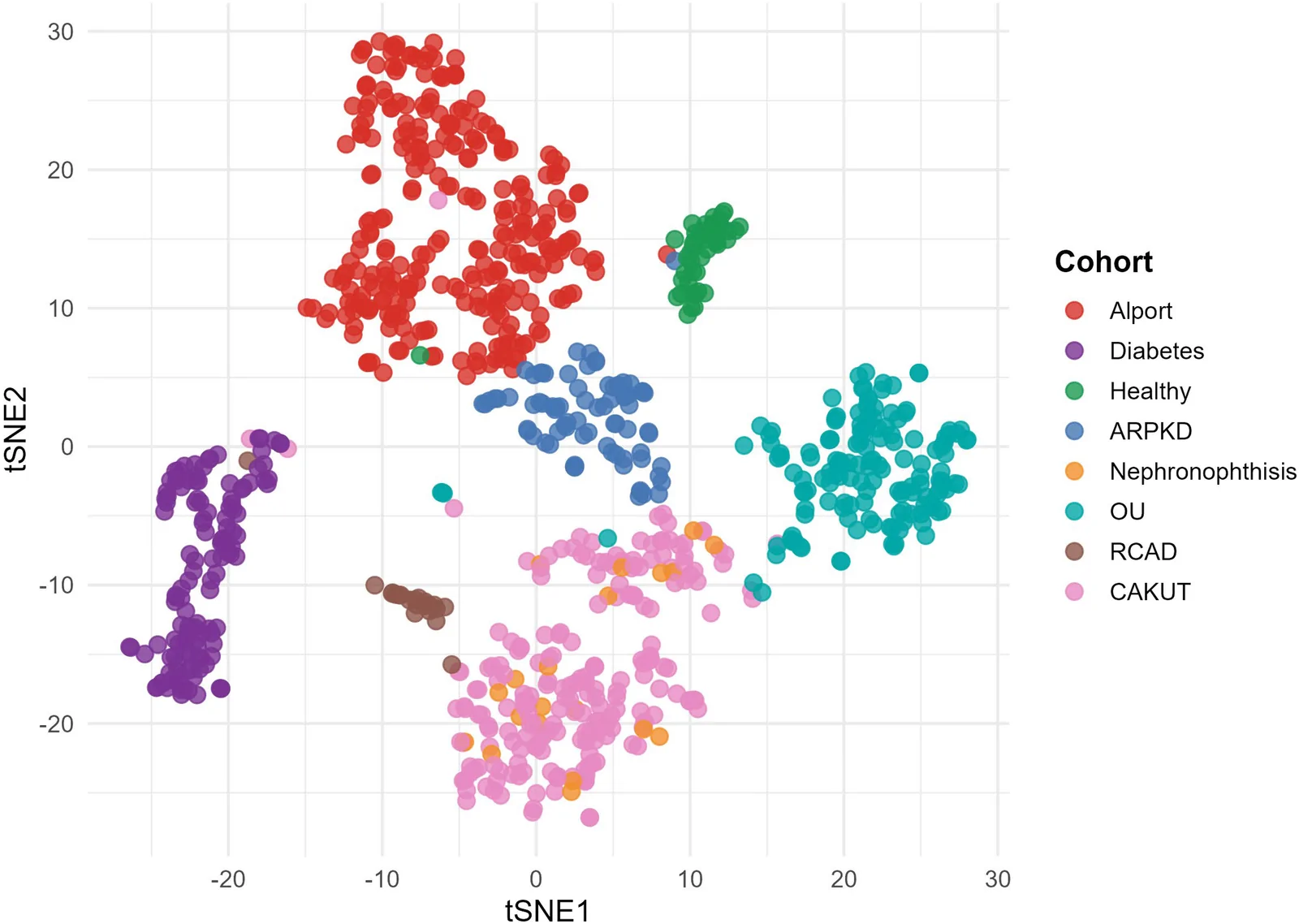
t-distributed stochastic neighbor embedding visualization of urinary peptide profiles across pediatric cohorts. The analysis demonstrates distinct clustering and molecular separation of Alport syndrome samples from other pediatric kidney diseases and healthy individuals according to global urinary peptidomic profiles. ARPKD, Autosomal Recessive Polycystic Kidney Disease; CAKUT, Congenital Anomalies of the Kidney and Urinary Tract; OU, obstructive uropathies; RCAD, Renal cysts and diabetes syndrome.

For the identification of urinary peptides associated with AS, a total of 661 samples from 471 pediatric patients were included in the discovery cohort and 272 samples from 199 pediatric patients in the independent validation cohort (Table 1). The discovery cohort was used for classifier construction and cross-validation (Figure 1). After pairwise comparisons between the cohorts, 151 peptides with significantly different abundance (false discovery rate < 0.05) and a consistent direction of change across all comparisons were identified.

Sequence information was available for 87 peptides corresponding to 36 proteins (Table 2). The sequenced peptides were used for support vector machine classifier construction and evaluation in the independent validation cohort (step 4 in Figure 1). Among them, 53 peptides (61%) were derived from collagen fragments. Overall, 63 peptides (72%) showed increased abundance and 24 peptides (28%) showed decreased abundance in AS compared with all other groups.

**Table 2:** Sequenced urinary peptides included in the pediatric Alport syndrome classifier

Protein name	Peptide sequence	Modifications	Abundance in Alport compared with other cohorts
Alpha-1-antitrypsin	TIDEKGTEAAGAMF	None	Increased
	FSNGADLSGVTEEAPLKL	None	Increased
Apolipoprotein A-IV	RRRVEPYGENF	None	Increased
	EKDLRDKVNSF	None	Increased
Basement membrane-specific heparan sulfate proteoglycan core protein	VEVGEAGQGKDF	None	Increased
Chloride intracellular channel protein 6	EAEARGTRGAHGETEAEEGAPEGAEVPQG	None	Decreased
Collagen alpha-1 (I) chain	DDGEAGKPGRP	1xOxidation [P]	Increased
	DDGEAGKPGRP	2xOxidation [P8; P11]	Increased
	GPPGPRGRTGDA	2xOxidation [P2; P3]	Increased
	GPPGEAGKPGEQG	2xOxidation [P3; P9]	Decreased
	APGDRGEPGPPGP	2xOxidation [P2; P8]	Increased
	PPGEAGKPGEQGVP	2xOxidation [P2; P8]	Increased
	PPGEAGKPGEQGVP	3xOxidation [P2; P8; P14]	Increased
	GPPGEAGKPGEQGVP	3xOxidation [P3; P9; P15]	Decreased
	GPPGKNGDDGEAGKPG	2xOxidation [P3; P15]	Decreased
	IGPPGPAGAPGDKGESGP	2xOxidation [P6; P]	Increased
	PPGPPGKNGDDGEAGKPG	2xOxidation [P]	Decreased
	TGPIGPPGPAGAPGDKGESGP	2xOxidation [P9; P]	Increased
	GKNGDDGEAGKPGRPGERGPPGP	2xOxidation [P]	Decreased
	ADGQPGAKGEPGDAGAKGDAGPPGPAGP	3xOxidation [P5; P11; P22]	Increased
	ERGSPGPAGPKGSPGEAGRPGEAGLPGAKG	4xOxidation [P10; P14; P20; P26]	Decreased
	LDGAKGDAGPAGPKGEPGSPGENGAPGQMGPRG	3xOxidation [P17; P20; P26]	Decreased
	RGAPGDRGEPGPPGPAGFAGPPGADGQPGAKGEP	2xOxidation [P22; P]	Decreased
	GEPGKAGERGVPGPPGAVGPAGKDGEAGAQGPPGP	3xOxidation [P12; P14; P]	Decreased
	NAGPPGPPGPAGKEGGKGPRGETGP	2xOxidation [P7; P]	Increased
	GFAGPPGADGQPGAKGEPGDAGAKGDAGPPGPA	3xOxidation [P12; P18; P29]	Decreased
	LDGAKGDAGPAGPKGEPGSPGENGAPGQMGPRG	4xOxidation [P13; P17; P20; P26]	Decreased
	GPPGPPGKNGDDGEAGKPGRPG	4xOxidation [P5; P6; P18; P21]	Decreased
Collagen alpha-1 (II) chain	PTGARGPEGAQGPRGEPGTPGSPGPAGASGNP	1xOxidation [P23]	Decreased
Collagen alpha-1 (III) chain	SPGERGETGPPGP	2xOxidation [P2; P10]	Increased
	DGESGRPGRPGER	2xOxidation [P7; P10]	Increased
	SPGPKGDKGEPGGPGADGVPG	1xOxidation [P]	Increased
	GEPGGKGERGAPGEKGEGGPPG	3xOxidation [P3; P12; P20]	Decreased
	PNGQGPQGPKGDPGPPGIPGR	3xOxidation [P15; P16; P]	Increased
	DGVPGKDGPRGPTGP	None	Increased
	SPGERGETGPPGP	1xOxidation [P11]	Increased
	GLPGTGGPPGENGKPG	2xOxidation [P3; P8]	Decreased
Collagen alpha-1 (IV) chain	RDGLPGPPGSPGPP	2xOxidation [P5; P7]	Decreased
Collagen alpha-1 (XI) chain	GQKGSKGDKGENGPPGPPGLQGPVGAPGIAG	1xOxidation [P27]	Increased
	QGLPGPPGEKGENGDVGPMGPPGPPGPRGPQGPNGADGPQGPPGSVG	3xOxidation [P4; P6; P7]	Decreased
Collagen alpha-1 (XIV) chain	KGNPGVGTQGPRGPPGPAG	2xOxidation [P11; P14]	Increased
Collagen alpha-1 (XVI) chain	EGPPGKQGFYGPPG	3xOxidation [P3; P4; P12]	Increased
Collagen alpha-1 (XVII) chain	PPGPPGPPGPPGSISSSGQ	1xOxidation [P]	Decreased
Collagen alpha-1 (XVIII) chain	TGQKGSLGEAGAPGH	1xOxidation [P13]	Increased
Collagen alpha-1 (XXIV) chain	PPGSQGPKGQRGSRGPDGL	2xOxidation [P1; P2]	Increased
Collagen alpha-2 (I) chain	EEGKRGPNGEAGS	None	Increased
	DGPPGRDGQPGHK	2xOxidation [P4; P10]	Increased
	SPGNIGPAGKEGPVG	1xOxidation [P2]	Increased
	PPGRDGQPGHKGER	2xOxidation [P2; P8]	Increased
	DGPPGRDGQPGHKGE	2xOxidation [P4; P10]	Increased
	DGPPGRDGQPGHKGER	2xOxidation [P4; P10]	Increased
Collagen alpha-2 (V) chain	GFPGSDGLPGPKGAQGERGPVGSSGPKGSQG	2xOxidation [P9; P]	Decreased
Collagen alpha-2 (XI) chain	PGERGAAGSGGPIGPP	3xOxidation [P1; P12; P15]	Decreased
	PAGSPGERGAAGSGGPIG	None	Increased
Collagen alpha-3 (V) chain	SPGSMGPRGDTGPAGPP	3xOxidation [P2; P7; P13]	Decreased
Collagen alpha-3 (IX) chain	GDLGRPGPKGTPGVAGPSGEPG	3xOxidation [P6; P8; P12]	Increased
Collagen alpha-4 (IV) chain	PGEPGPPGDDGFPGERGDKGTPG	2xOxidation [P13; P22]	Increased
Collagen alpha-6 (IV) chain	PPGEPGPPGPW	3xOxidation [P2; P5; P7]	Increased
	GPPGPPGPSSNQG	2xOxidation [P3; P5]	Decreased
Complement C3	ITHRIHWESASL	None	Increased
Epidermal growth factor receptor kinase substrate 8-like protein 2	WGPASPTHKLP	None	Increased
Fibrinogen alpha chain	EGTHSTKRG	None	Increased
	DHEGTHSTKR	None	Increased
	DHEGTHSTKRG	None	Increased
	SRGSESGIFTNTK	None	Increased
	HEGTHSTKRG	None	Increased
Hemoglobin subunit alpha	HLPAEFTPAVHASLDK	None	Increased
	GKVGAHAGEYGAEALERM	None	Increased
	VKAAWGKVGAHAGEYGAEALERM	None	Increased
	DKTNVKAAWGKVGAHAGEYGAEALERM	1xOxidation [M27]	Increased
	SPADKTNVKAAWGKVGAHAGEYGAEALER	None	Increased
	SPADKTNVKAAWGKVGAHAGEYGAEALERM	1xOxidation [M30]	Increased
	PHFDLSHGSAQVK	None	Increased
Hemoglobin subunit beta	GKVNVDEVGGEALGRL	None	Increased
	AHHFGKEFTPPVQAA	None	Increased
Hemoglobin subunit delta	VVAGVANALAHKYH	None	Increased
Immunoglobulin lambda-like polypeptide 5	SLTPEQWKSH	None	Increased
Integrin alpha-7	EPPEQQEPGERQEP	None	Decreased
Inter-alpha-trypsin inhibitor heavy chain H4	RDTDRFSSHVGGTLGQF	None	Increased
Pigment epithelium-derived factor	VTKFDSRKTSLEDF	None	Increased
Protein AMBP	YGRAPQLRET	None	Increased
Retinol-binding protein 4	SRDPNGLPPEAQK	None	Increased
RNA-binding protein 20	ATAGFYEYGKASSGQ	None	Increased
Thymosin beta-4	SDKPDMAEIEKFDKSKL	1xAcetylation [N-Term]	Increased
Uromodulin	VIDQSRVLNLGPITR	None	Increased
	SGSVIDQSRVLNLGPIT	None	Increased
	SVIDQSRVLNLGPITR	None	Increased

In the cross-validated discovery cohort, the classifier demonstrated excellent performance, achieving an AUC of 0.997 (95% CI, 0.995–0.999) (Figure 3a). The selected peptides enabled accurate discrimination between patients with AS and the other study cohorts, with significantly higher classifier scores (*P* < 0.001) observed in the AS cohort compared with the other study cohorts (Figure 3b). Based on the cross-validated discovery scores, the optimal classification threshold was determined using the Youden index at −0.196.

**Figure 3:**
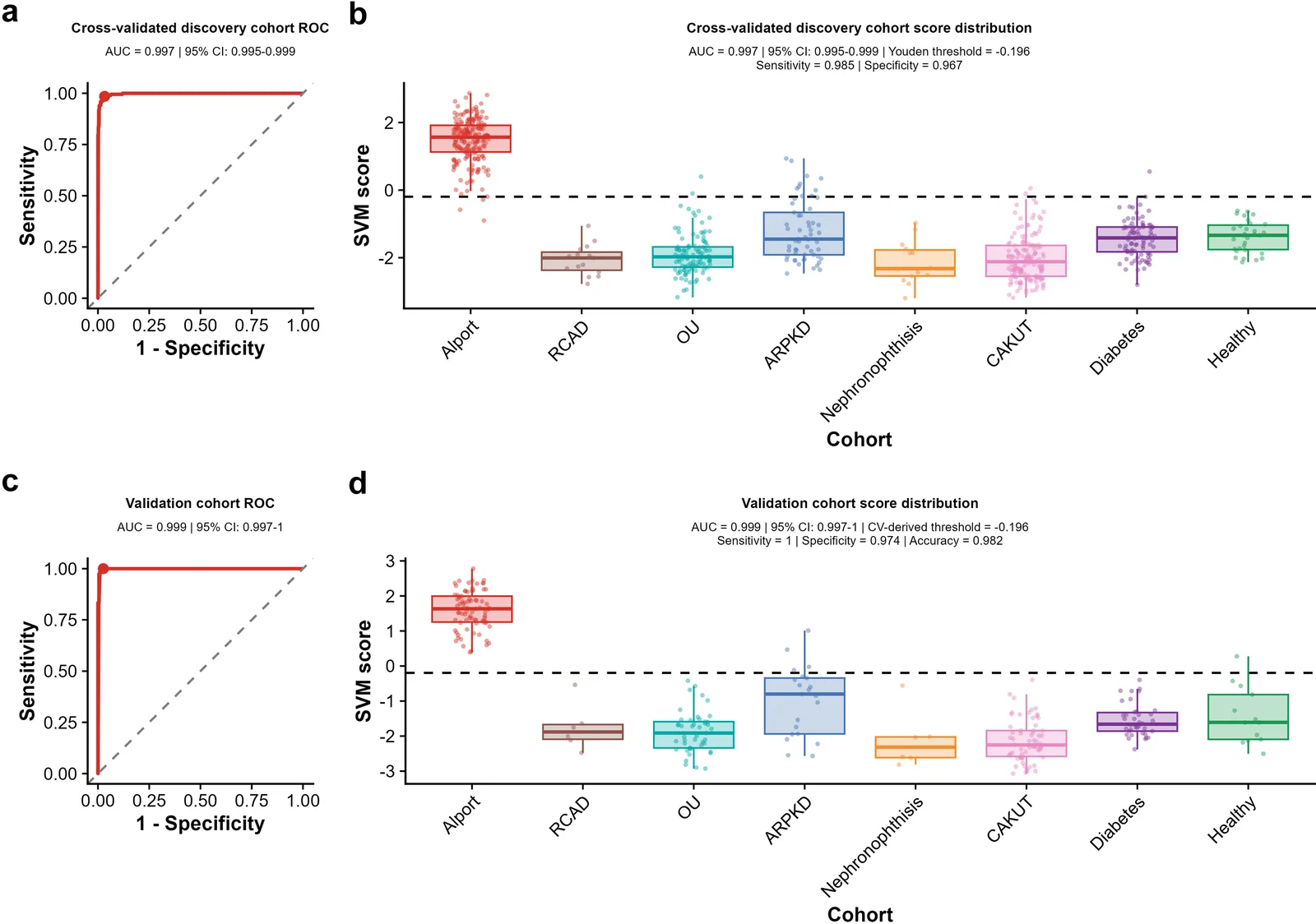
Performance of the pediatric urinary peptidomic classifier for the non-invasive diagnosis of Alport syndrome in the cross-validated discovery and independent validation cohorts. (a, c) Receiver operating characteristic curves demonstrating classifier performance in the cross-validated discovery and independent validation cohorts, respectively. (b, d) Distribution of classifier scores across Alport syndrome, healthy individuals, and non– Alport syndrome kidney disease cohorts in the cross-validated discovery and validation cohorts, respectively, shown as boxplots with individual sample points. The dashed horizontal line represents the classification threshold determined using the Youden index from the cross-validated discovery cohort and applied unchanged to the independent validation cohort. In both cohorts, classifier scores were significantly higher in patients with AS than in all comparator cohorts (all *P* < 0.001). Classifier performance metrics, including area under the ROC curve (AUC), sensitivity, specificity, and accuracy, are indicated within the panels. ARPKD, Autosomal Recessive Polycystic Kidney Disease; AUC, area under the curve; CAKUT, Congenital Anomalies of the Kidney and Urinary Tract; CI, confidence interval; OU, obstructive uropathies; RCAD, Renal cysts and diabetes syndrome; ROC, receiver operating characteristic.

### Validation of the Classifier

Application of this predefined threshold to the independent validation cohort confirmed the robustness of the classifier. In the validation dataset, the classifier achieved an AUC of 0.999 (95% CI, 0.997–1.000) (Figure 3c), with 100% sensitivity, 97.4% specificity, and 98.2% accuracy. Consistently, classifier scores remained significantly higher *(P* < 0.001) in patients with AS compared with the other cohorts (Figure 3d).

### Influence of Demographic and Clinical Variables

After adjustment for age, sex, and eGFR, classifier scores remained higher in patients with AS compared with non–AS cohorts. The age-, sex-, and eGFR-adjusted classifier score retained strong discriminatory performance in the validation set, with an adjusted AUC of 0.918 (95% CI, 0.884-0.951).

### Evaluation of the Classifier Over Time

As longitudinal follow-up samples were available for the AS cohort, we assessed whether classifier scores remained stable across consecutive visits in the 17 patients with AS from the validation cohort (Figure 4), in whom classifier performance had already been evaluated. Overall, classifier scores were consistent over time within individual patients and remained above the predefined threshold throughout follow-up. The classifier also classified as positive, with 1 patient carrying a hemizygous *COL4A5* variant whose pathogenicity classification had remained inconclusive^12^ (Figure 4).

**Figure 4:**
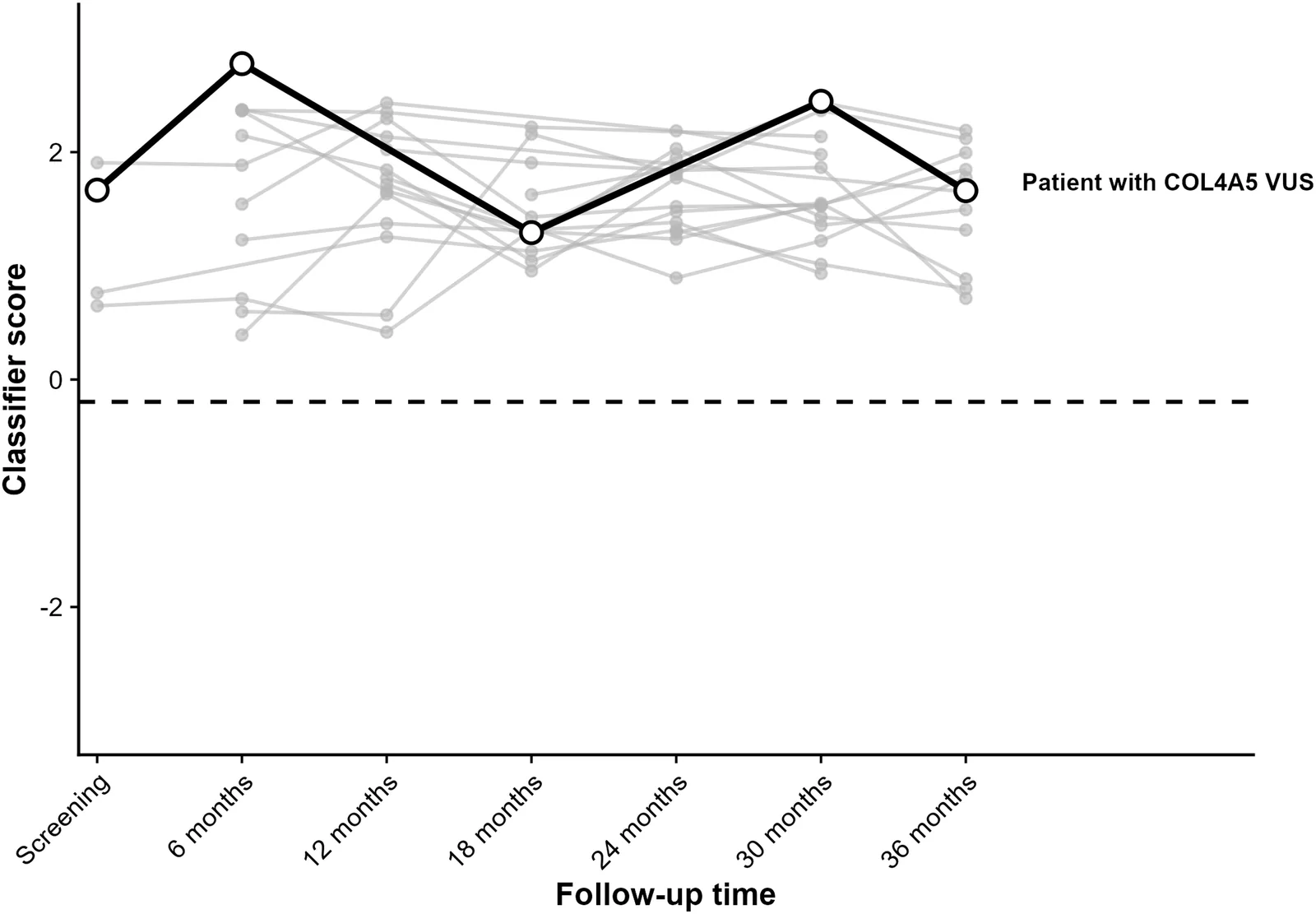
Longitudinal assessment of urinary peptidomic classifier scores in pediatric Alport syndrome patients during follow-up in the validation cohort. Each line represents repeated urine samples obtained from an individual patient across multiple follow-up visits. The dashed horizontal line indicates the predefined diagnostic threshold established in the discovery cohort. All patients consistently remained above the diagnostic threshold throughout follow-up, supporting the temporal stability and robustness of the identified urinary peptidomic signature over time. The trajectory of the patient carrying a hemizygous *COL4A5* variant of uncertain significance (VUS) is highlighted in black and remained consistently above the diagnostic threshold across all available visits.

Additionally, the effects of ACEi treatment and disease progression on classifier scores were evaluated using repeated urine samples collected during follow-up. Longitudinal analyses included 81 urine samples from 17 pediatric patients. For progression analyses, 43 samples from 9 nonprogressors and 38 samples from 8 progressors were available. In linear mixed-effects models accounting for repeated measurements within individuals, classifier scores were not significantly associated with disease progression (*P* = 0.79). Similarly, no significant association between ACEi treatment and classifier score was observed (*P* = 0.66), although interpretation of this analysis was limited by the small number of observations obtained before treatment initiation (3 visits without ACEi treatment vs. 78 visits during ACEi treatment).

## Discussion

The present study describes the development and validation of the first disease-specific urinary peptidomic classifier for the diagnosis of pediatric AS. The classifier demonstrated excellent diagnostic performance in the validation cohort despite the marked heterogeneity of the included pediatric populations. The very high performance likely reflects the strong molecular distinctiveness of early CKD-stages in pediatric AS relative to the other heterogeneous cohorts included in this study. Importantly, classifier scores appeared stable across longitudinal follow-up samples and were not significantly associated with progression status during follow-up, suggesting that the identified peptidomic signature reflects underlying disease biology rather than transient clinical fluctuations. Moreover, the classifier retained strong discriminatory performance after adjustment for age, sex, and eGFR, indicating that its performance was not solely driven by differences in kidney function or demographic characteristics between the cohorts.

A major biological finding of this study was the identification of a pediatric AS-associated urinary peptidomic signature and the characterization of its underlying molecular composition. The identified classifier included peptides derived from multiple proteins, including basement membrane-associated collagen IV chains as well as fibrillar and extracellular matrix-associated collagens. This observation is highly consistent with the pathophysiology of AS, in which pathogenic variants in *COL4A3*, *COL4A4*, or *COL4A5* lead to progressive abnormalities of the glomerular basement membrane, extracellular matrix remodeling, and disruption of collagen network organization. Interestingly, both increased and decreased abundance patterns were observed among collagen fragments, potentially reflecting complex and heterogeneous extracellular matrix turnover processes associated with disease progression and tissue remodeling in AS, as previously suggested for collagen fragmentation patterns in CKD.^24^

Beyond collagen-derived peptides, the classifier also contained peptides originating from uromodulin, complement C3, and basement membrane-specific heparan sulfate proteoglycan, indicating that tubular injury, inflammatory activation, and basement membrane remodeling may also contribute to the molecular signature of pediatric AS. These findings are consistent with previous reports of early tubulointerstitial stress in pediatric AS and with recent studies highlighting extracellular matrix remodeling and collagen turnover as central mechanisms in AS pathophysiology, involving not only the glomerular basement membrane but also the tubulointerstitial compartment.^25^^,^^26^ The heterogeneous molecular composition of the identified classifier further supports the concept that multimarker molecular approaches may provide superior characterization of AS-related kidney injury compared with single biomarker strategies, given the multiple pathophysiological pathways involved in disease progression.^27^

The overlap between the identified pediatric AS signature and previously reported adult AS urinary peptidomic signature was minimal.^28^ Among the 419 peptides previously described as AS-associated in adults, only 1 peptide overlapped with the pediatric classifier, whereas no overlap was observed with the final Alport178 classifier. This limited overlap likely reflects multiple contributing factors. First, age-related differences in the urinary peptidome have been described previously,^29^ with pediatric profiles presumably being less influenced by accumulated comorbidities and treatment exposure than those of adult patients. This may be particularly relevant given that the 2 cohorts also differed in their genetic distribution (predominantly *COL4A5* variants in the pediatric cohort vs. *COL4A3/COL4A4* variants in the adult cohort). Although the downstream pathomechanism converges on the α3α4α5 collagen IV network, autosomal forms tend to manifest later in life and therefore may be more susceptible to modulation by factors such as disease stage, treatment, cardiovascular comorbidities, and environmental exposures, which are likely to be more variable in the adult cohort and could affect the urinary peptidome. An additional explanation for the limited overlap may be differences in disease stage. In children, urinary peptide patterns may more closely reflect primary alterations in collagen IV, whereas in adults they may increasingly reflect fibrosis and broader extracellular-matrix remodeling associated with chronic kidney injury. These later-stage changes may overlap with other chronic kidney diseases and influence the features selected for classification. Therefore, the pediatric and adult classifiers should be considered age-specific until their performance is evaluated across age groups.

Several limitations of this study should be acknowledged. Although the number of urine samples was substantial, the number of pediatric patients with AS remained relatively small, and the cohort was predominantly composed of patients with X-linked disease. This distribution reflects the expected prevalence of the different genetic forms of AS in pediatric populations, where X-linked disease accounts for most of the cases, thereby supporting the representativeness of the cohort. In addition, the limited number of genetically unresolved cases did not allow separate assessment of classifier performance in this subgroup; therefore, dedicated validation in patients with negative or inconclusive molecular findings is required. The inclusion of healthy controls in the comparator population may have contributed to optimistic estimates of overall classifier performance, although most of the comparator samples were derived from children with kidney disease or diabetes. Moreover, no fully external validation cohort was available beyond the datasets included in this study. Nevertheless, the validation cohort was analyzed in a completely blinded manner, and none of its samples were used during the discovery phase. Furthermore, longitudinal analyses were limited by the relatively small number of patients with repeated follow-up samples. In particular, evaluation of the potential impact of ACEi treatment was constrained by the low number of observations obtained before treatment initiation, which limited the statistical power to detect treatment-related differences in classifier scores.

Despite these limitations, the developed classifier may have important clinical implications. The diagnostic value of urinary peptidomics may be particularly relevant in clinically ambiguous cases, such as patients with variants of uncertain significance, isolated heterozygous *COL4A3/COL4A4* variants of unclear penetrance, or discrepancies between genetic and clinical findings.

In our study, the classifier consistently identified as positive a patient carrying a hemizygous *COL4A5* variant of uncertain significance whose pathogenicity classification had remained inconclusive. The variant was classified as a variant of uncertain significance according to the American College of Medical Genetics and Genomics criteria but as likely pathogenic in ClinVar.^12^ In the clinical context of early manifestations consistent with AS, including microalbuminuria, together with a family history of early kidney failure in a relative at the age of 19 years, the variant was considered highly likely to be disease-causing. This case illustrates a potential clinical application of urinary peptidomics in situations where genetic findings remain inconclusive despite a clinical presentation suggestive of AS. The persistently elevated classifier scores observed across longitudinal follow-up visits suggest that the urinary peptide profile of this patient shared molecular characteristics with genetically confirmed AS cases. Although limited to a single observation, this finding further supports the potential role of urinary peptidomics as an adjunct to genetic testing in the diagnostic evaluation of suspected AS.

Future studies should focus on external multicenter validation of the classifier and evaluation of its clinical utility in patients with negative or inconclusive genetic testing results. In addition, further studies are needed to determine whether urinary peptidomic signatures may also provide prognostic information or support disease monitoring in pediatric AS.

In conclusion, a validated and highly accurate urinary peptidomic classifier was developed that may be used for the noninvasive diagnosis of pediatric AS. The identified signature may complement genetic testing and support earlier recognition of AS, particularly in clinically suspected cases with inconclusive molecular findings.

## Disclosure

AO has received consultancy or speaker fees or travel support from Astellas, Astrazeneca, Bioporto, Boehringer Ingelheim, Fresenius Medical Care, GSK, Bayer, Sanofi-Genzyme, Sobi, Menarini, Lilly, Chiesi, Otsuka, Novo-Nordisk, Sysmex and CSL-Vifor and Spafarma. HM is the founder and co-owner of Mosaiques Diagnostics. JS and MK are employed by Mosaiques Diagnostics. All other authors declare no conflict of interest related to this manuscript.

## Supplementary Material

Supplementary File (PDF) **Supplementary Methods. Supplementary References. Table S1.** Clinical and demographic characteristics of the study cohorts included in the urinary peptidomic analysis.

## Footnotes

Supplementary File (PDF)

**Supplementary Methods.**

**Supplementary References.**
**Table S1.** Clinical and demographic characteristics of the study cohorts included in the urinary peptidomic analysis.
