# Supplementary material for: Urinary Peptidomic Signature for Noninvasive Diagnosis of Alport Syndrome in Children
Source: Kidney Int Rep. 2026 Sep 25;11(12):107093. doi: 10.1016/j.ekir.2026.107093 (PMC13695312; doi:10.1016/j.ekir.2026.107093)
Supplement: Supplementary file 1 [file mmc1.pdf]

# Supplementary Material

## Table of Contents

### Supplementary Methods

1. Urine sample preparation for CE–MS
2. CE–MS analysis
3. CE–MS data processing, calibration and normalization
4. Peptide annotation and database storage
5. Sequencing of peptides

### Supplementary Tables

**Supplementary Table S1**. Clinical and demographic characteristics of the study cohorts included in the urinary peptidomic analysis.

### Supplementary References

References

**Supplementary Methods**

1. **Urine sample preparation for CE–MS**

Frozen urine aliquots were thawed and diluted in a 1:1 ratio with a solution containing 2 M urea, 10 mM $NH_4OH$, and 0.02% SDS (700 µl urine + 700 µl buffer). High-molecular-weight proteins were removed by ultrafiltration using a 20 kDa molecular weight cut-off centrifugal filter device (Centristat; Sartorius, Göttingen, Germany). The obtained filtrate was then desalted and enriched for peptides using PD-10 gel filtration columns (GE Healthcare Bio-Sciences, Uppsala, Sweden), allowing the removal of urea, salts, and other low-molecular-weight contaminants. Following desalting, samples were lyophilized and stored at 4°C until further analysis. Prior to CE–MS analysis, dried samples were reconstituted in 10 µl of HPLC-grade water.

2. **CE–MS analysis**

For CE–MS measurements, approximately 280 nl of sample was injected into the capillary under pressure (2 psi for 99 s). Peptide separation was performed using a P/ACE MDQ capillary electrophoresis system (Beckman Coulter, Fullerton, CA, USA) coupled online to a MicrOTOF II mass spectrometer (Bruker Daltonics, Bremen, Germany). The separation electrolyte consisted of 20% acetonitrile and 0.94% formic acid in HPLC-grade water. Electrospray ionization was achieved using an interface supplied by Agilent Technologies (Palo Alto, CA, USA) operated at a spray voltage between −4.0 and −4.5 kV. Mass spectra were collected across an m/z range of 350–3000, with spectra accumulated every 3 s.

3. **CE–MS data processing, calibration and normalization**

Raw CE–MS data were processed using MosaFinder software. Signals corresponding to different charge states of the same peptide were deconvoluted and combined into a single neutral molecular mass, following previously established procedures [16]. Only peptide features exhibiting a charge state greater than one ($z > 1$) and detected in at least three consecutive spectra were included in subsequent analyses. Each peptide feature was characterized by its molecular mass and capillary electrophoresis migration time.

Calibration of molecular mass and migration time was performed using 3151 endogenous reference signals. Molecular mass calibration employed global linear regression, whereas migration time calibration was based on local linear regression. To reduce analytical variability between samples, signal intensities were normalized by linear regression using a panel of 29 highly abundant endogenous peptides serving as internal standards [S1].

### 4. Peptide annotation and database storage

Following calibration and normalization, each detected peptide was defined by three parameters: calibrated molecular mass (Da), calibrated migration time (min), and normalized signal intensity. Peptide features from all samples were subsequently aligned across runs, annotated, and stored within a Microsoft SQL database, enabling comparative analyses and large-scale cohort evaluations.

### 5. Sequencing of peptides

Candidate biomarker peptides were identified by tandem mass spectrometry using either CE–MS/MS or LC–MS/MS approaches. Analyses were performed on an Ultimate 3000 nano-flow liquid chromatography system (Dionex/LC Packings, USA) or a P/ACE MDQ capillary electrophoresis platform (Beckman Coulter, Fullerton, CA, USA), both coupled to a Q Exactive™ Plus Hybrid Quadrupole–Orbitrap™ mass spectrometer (Thermo Fisher Scientific, Waltham, MA, USA). The instrument operated in data-dependent acquisition mode, alternating automatically between full MS scans and MS/MS fragmentation events. Survey spectra were acquired in the Orbitrap over an m/z range of 300–2000, after which selected precursor ions were isolated and fragmented for sequence determination.

MS/MS data were searched against the human UniProt non-redundant protein database using Proteome Discoverer (v2.4) with the SEQUEST HT search algorithm. No fixed modifications were specified, whereas oxidation of methionine and proline residues was included as a variable modification. Searches were performed using a precursor mass range of 800–8000 Da, with precursor and fragment mass tolerances set to 5 ppm and 0.05 Da, respectively. Peptide identifications were considered high confidence according to the Xcorr-based criteria implemented in Proteome Discoverer. To further improve assignment reliability, experimentally observed CE migration times were compared with

migration times predicted from peptide sequence-derived charge properties at approximately pH 2, thereby reducing the likelihood of false-positive identifications [S2].

## Supplementary Tables

**Supplementary Table S1.** Clinical and demographic characteristics of the study cohorts included in the urinary peptidomic analysis.

| Characteristics | Alport (*n* = 282)[1] | CAKUT (*n* = 209) | OU (*n* = 151) | Diabetes (*n* = 128) | ARPKD (*n* = 78)[2] | Healthy (*n* = 44) | RCAD (*n* = 21) | Nephronophthisis (*n* = 20) |
|---|---|---|---|---|---|---|---|---|
| Age, years, mean (SD) | 10 (4) | 12 (3) | 8 (5) | 10 (4) | 7 (4) | 10 (5) | 6 (4) | 12 (3) |
| Female, % | 4 | 29 | 13 | 52 | 53 | 36 | 24 | 40 |
| eGFR (mL/min/1.73 $m^2$), mean (SD) | 128 (31) | 30 (12) | 49 (18) | 134 (23) | 69 (35) | n.a. | 115 (19) | 28 (9) |

CAKUT: Congenital Anomalies of the Kidney and Urinary Tract; OU: obstructive uropathies; ARPKD: Autosomal Recessive Polycystic Kidney Disease; RCAD: Renal cysts and diabetes syndrome.

n.a.: not applicable.

[1] 282 samples/57 patients

[2] 78 samples/40 patients

**Supplementary References**

[S1] Jantos-Siwy J, Schiffer E, Brand K, et al. Quantitative urinary proteome analysis for biomarker evaluation in chronic kidney disease. *J Proteome Res.* 2009;8(1):268-281. doi:10.1021/pr800401m

[S2] Zürbig P, Renfrow MB, Schiffer E, et al. Biomarker discovery by CE-MS enables sequence analysis via MS/MS with platform-independent separation. *Electrophoresis.* 2006;27(11):2111-2125. doi:10.1002/elps.200500827
